# Fractionated Concurrent Exercise throughout the Day Does Not Promote Acute Blood Pressure Benefits in Hypertensive Middle-aged Women

**DOI:** 10.3389/fcvm.2017.00006

**Published:** 2017-02-14

**Authors:** Luan M. Azevêdo, Alice C. de Souza, Laiza Ellen S. Santos, Rodrigo Miguel dos Santos, Manuella O. M. de Fernandes, Jeeser A. Almeida, Emerson Pardono

**Affiliations:** ^1^Programa de Pós Graduação em Educação Física (PPGEF), Universidade Federal de Sergipe, São Cristóvão, Brazil; ^2^Programa de Pós Graduação em Saúde e Desenvolvimento na Região Centro-Oeste (PPGSD), Universidade Federal de Mato Grosso do Sul, Campo Grande, Brazil

**Keywords:** systemic arterial hypertension, cardiovascular diseases, combined exercise, post-exercise hypotension, cardioprotection

## Abstract

Hypertension is a chronic disease that affects about 30% of the world’s population, and the physical exercise plays an important role on its non-pharmacological treatment. Anywise, the *dose*–*response* of physical exercise fractionation throughout the day demands more investigation, allowing new exercise prescription possibilities. Therefore, this study aimed to analyze the acute blood pressure (BP) kinetics after 1 h of exercises and the BP reactivity after different concurrent exercise (CE) sessions and its fractioning of hypertensive middle-aged women. In this way, 11 hypertensive women voluntarily underwent three experimental sessions and one control day [control session (CS)]. In the morning session (MS) and night session (NS), the exercise was fully realized in the morning and evening, respectively. For the fractionized session (FS), 50% of the volume was applied in the morning and the remaining 50% during the evening. The MS provided the greatest moments (*p* ≤ 0.05) of post-exercise hypotension (PEH) for systolic BP (SBP) and highest reduction of BP reactivity for SBP (~44%) and diastolic BP (DBP) (~59%) compared to CS (*p* ≤ 0.05). The findings of the present study have shown that MS is effective for PEH to SBP, as well as it promotes high quality of attenuation for BP reactivity, greater than the other sessions.

## Introduction

World data have shown that systemic arterial hypertension (SAH) has one of the largest number of occurrences (about 30%), and it was indirectly responsible for about 9.4 million deaths in 2010 (1–3). The SAH is understood as a clinical condition caused by multiple factors, showing high and sustained levels of blood pressure (BP) for systolic (SBP ≥ 140 mmHg) and/or diastolic (DBP ≥ 90 mmHg).

There are some possibilities for SAH treatment, including non-pharmacological therapy. It includes changes in behavioral factors, among which physical exercise presents one of the most effective and safe intervention (4). The acute benefits after exercise include post-exercise hypotension (PEH) and the attenuation of BP reactivity, as the morphological and functional adaptations when realized chronically, increasing the basal metabolism and the reduction of the rest BP values (5–7).

Post-exercise hypotension is termed by the post-exercise period in which are registered lower BP values than those measured in the pre-exercise or in a control day (8, 9). This phenomenon has great clinical relevance (10–12) and has been shown for normotensive (13), pre-hypertensive (14), and hypertensive (15) individuals, and it can be observed during the 24 h following the exercise (14, 16). These BP decay ranged approximately −3.5/−2.5 mmHg after aerobic exercise (AE) and −1.8/−3.2 mmHg after dynamic resistance exercise (RE) (6), and the most important, it is enough to decrease by 4% of overall risk of mortality caused by coronary diseases (17).

Some studies (18–20) suggested that AE may be prioritized in the prescription programs for hypertensive individuals since it promotes better PEH than RE (18). However, the effect of AE and RE combination in the same session or even on separate days [concurrent exercise (CE)] (19, 21) has also been studied.

Since 2004, many studies have analyzed the BP kinetics after CE (18, 22–30), but only five of them were conducted with elderly population (22, 23, 27–29). Additionally, just few researchers evaluated this response at different day shifts (31–34) and have examined the fractionation volume of a workout session, performing it throughout the day (14, 35–39).

To the best of our knowledge, this was the first study to investigate the effect of a CE session performed in the morning, evening, and fractionated sessions (50% in the morning and the other 50% at night) and evaluating the acute BP kinetics and its reactivity after sessions in hypertensive middle-aged women. Therefore, this study aimed to analyze the acute BP kinetics after 1 h of exercises and the BP reactivity after different CE sessions and its fractioning of hypertensive middle-aged women. The hypothesis of our study was that the CE session fractionized throughout the day would promote better PEH than the other sessions.

## Materials and Methods

### Experimental Approach to the Problem

All sessions, exercise, and control were performed in common time for all volunteers (08:00 h and 18:00 h), as well as randomized and with a minimum interval of 72 h between each, to obtain uniformity and minimize bias that could influence BP kinetics regarding the different day times. Moreover, all experimental sessions were conducted by three qualified professionals, yielding a ratio of 3:2 (professional:participant) at all times during the study.

The volunteers were instructed to maintain a moderate rate of movement during the RE (2 s for the concentric phase and 2 s for the eccentric phase), breathing freely to avoid Valsalva maneuver and ingest water *ad libitum* during all sessions. Additionally, the intake of foods/drinks containing caffeine was not suspended, because some studies (40–42) have shown that suspending the chronic use of this substance leads to various side effects that could affect our results, besides causing possible discomfort to volunteers.

### Subjects

Eleven hypertensive middle-aged women (57.5 ± 5.1 years) voluntarily participated of this study and had at least 3 months of CE experience. The anthropometric and hemodynamics characteristics of the study group are presented in Table 1; no differences were detected between the rest values over the days of sessions.

**Table 1:** **Anthropometric and hemodynamics characteristics of the study sample (*n* = 11)**.

	Mean ± SD	CI (95%)
**Anthropometrics**		
Age (years)	57.5 ± 5.1	54.0–60.9
Body mass (kg)	70.1 ± 10.6	62.9–77.2
Height (m)	1.5 ± 0.6	1.5–1.6
BMI (kg/m^2^)	30.7 ± 3.9	28.1–33.4
AC (cm)	98.8 ± 12.2	90.5–107.0
WC (cm)	90.4 ± 11.9	82.4–98.3
HC (cm)	102.4 ± 9.3	95.8–109.1
WHR	0.9 ± 0.1	0.8–1.0
**Hemodynamics^a^**		
SBP (mmHg)	121 ± 6.4	117–125
DBP (mmHg)	75 ± 7.1	70–80
MAP (mmHg)	89 ± 7.0	84–94
HR (bpm)	70 ± 8.6	64–76
DP (mmHg × bpm)	8,404 ± 975.2	7,749–9,059

*BMI, body mass index; AC, abdominal circumference; WC, waist circumference; HC, hip circumference; WHR, waist/hip ratio; SBP, systolic blood pressure; DBP, diastolic blood pressure; MAP, mean arterial pressure; HR, heart rate; DP, double product*.

*^a^All volunteers were under antihypertensive treatment*.

The present study has been approved by the Research Ethics Committee of Federal University of Sergipe (CAAE: 49154515.2.0000.5546) and followed the norms advocated by the Declaration of Helsinki (43). Additionally, all volunteers have signed an Informed Consent Form.

### Procedures

#### Anthropometric Assessment

The body mass (kg) and height (cm) of all volunteers were obtained using an anthropometric balance (Welmy SA, Santa Barbara do Oeste, Brazil), with a maximum capacity of 150 kg, and 0.1 kg of scale. From these two variables, the body mass index (BMI) was estimated.

Subsequently, the abdominal circumference (AC), waist circumference (WC), and hip circumference (HC) were measured using an anthropometric metal tape (Sanny, São Bernardo do Campo, Brazil) with 0.1 mm scale. With these values, the waist–hip ratio of all volunteers was estimated. All measurements were made following the World Health Organization protocol (44).

#### Subjective Perception of Effort (SPE)

The intensity of the exercises was estimated and controlled using the SPE of each participant, which was obtained from the visual analog scale OMNI-GSE (45). This instrument has high reliability in obtaining this variable, especially because it has facial expressions attached to the scale (score 0–10), that help recognize the effort that the individual is performing.

#### Eight Repetition Maximum Test (8-RM)

After three familiarization sessions with the exercises and following the proposed protocol, the volunteers underwent a test for estimation of the maximum loads, applied in 2 days and with a 72 h minimum interval between each of them. This interval allows to obtain greater reliability and reproducibility of results (46, 47). The reproducibility was estimated from the analysis of the intraclass correlation coefficient (ICC) between these two measures, adopting an ICC ≥0.90 as acceptable.

This test consisted in an execution of eight maximum repetitions (48), after preheating (1 × 10 repetitions at 50% load used in familiarization). The volunteer could not perform the ninth repetition, or either self-reported an SPE ≥9 and/or neuromuscular fatigue (inability to sustain the cadence of the movement). The load used to achieve this fatigue was adopted as the maximum load of each volunteer (100%), from which 75% of it was calculated to be used in the experimental protocols described later.

Each volunteer performed three attempts for the following exercises: Leg Press, Machine Row, Deadlift, and Bench Press, with a two to five rest-minutes period between them. These exercises were performed in alternating body segments order to facilitate recovery and minimize fatigue. It is noteworthy that prior standardized instructions and verbal encouragement were given in order to obtain results as close as possible to their maximum (49).

#### BP and BP Reactivity Assessment

All BP measurements were performed on the left arm having the volunteer comfortably sat in a quiet room at the controlled temperature of 25°C, using an automatic, calibrated and validated device (Microlife, model BP 3AC1-1) (50), by which it was obtained: systolic blood pressure (SBP), diastolic blood pressure (DBP), and heart rate.

Once the volunteers arrived at the laboratory, they were instructed to sit, and the BP was measured over 20 min, measuring the BP each 5 min in order to get the arithmetic mean of BP at rest. After this period, the sessions were conducted.

At the end of each session, the volunteers were led to a rest room in the laboratory, and they were instructed to remain sat for 1 h. During this time, the BP was measured every 15 min (Rec15, Rec30, Rec45, and Rec60). Finally, the volunteers underwent the cold pressor test (CPT) to analyze the BP reactivity.

The BP reactivity is a hemodynamic variable characterized by sudden BP increase after a stressful event of physical or psychological nature, and some studies involving physical exercise (7, 51) emulate such circumstances by applying a procedure known as CPT. The CPT consists on submerging one hand in ice water (~4°C), measuring up the BP in the contralateral arm immediately after 1 min of this submersion, as described by Hines and Brown (52).

#### Morning and Night Exercise Sessions

The morning and night sessions consisted of three sets of 10 repetitions (3 × 10 at 75% of 8-RM) with active rest between sets (1 min of moderate walk at 5–6 SPE scale), since this recover is suggested to enhance the PEH (53). The following exercises were Leg Press, Machine Row, Deadlift, and Bench Press. After the RE, the volunteers performed the AE (20 min of moderate-to-severe cycling at 7–8 SPE scale). The control session was conducted under the same conditions of this experimental session, with the exception that the subjects did not perform exercises.

#### Fractionized Exercise Session

The fractionized session (FS) was performed by the fragmentation of the proposed exercises before described. Thus, the volunteers reported to the laboratory in both shifts, in the morning (0800 hours) and at night (1800 hours) of the same day.

Once the volunteers arrived in the morning, the BP at rest was measured following the procedures described previously. After that, they performed three sets of 10 repetitions (3 × 10 at 75% of 8-RM) in the Leg Press and Machine Row, with active rest between sets (1 min of moderate walk at 5–6 SPE scale), plus 10 min of cycling at moderate–severe intensity (at 7–8 SPE scale). Subsequently, the volunteers were released to their respective daily activities.

When the volunteers returned to the laboratory at night, they performed three sets of 10 repetitions (3 × 10 at 75% of 8-RM) in the Deadlift and Bench Press, with active rest between sets (1 min of moderate walk at 5–6 SPE scale), plus 10 min of cycling at moderate–severe intensity (at 7–8 SPE scale). After these procedures, the volunteers were conducted to the rest room to record BP of 1 h post-exercise, and then they underwent the CPT.

#### Statistical Analysis

The results were expressed using elements of descriptive statistics (mean, SD, absolute and relative frequencies) for all values obtained. Data normality was tested from the Shapiro–Wilk test and the homogeneity by the Levene test.

Analysis of variance with repeated measures was used to compare the delta variations [BP_rest_ − (Rec15 or Rec30 or Rec45 or Rec60 or CPT)], adjusting for the main effect of Bonferroni. As for possible differences between sessions, the analysis of covariance, with multiple comparisons between pairs of Bonferroni, was applied.

All analyses were adjusted from the inclusion of antihypertensive drugs categorized as dichotomous covariates (54), as well the cardiovascular risk factors, such as BMI and menopause status in their respective categories, and smoking, diabetes, and hypercholesterolemia also as dichotomous covariates.

The power of the sample size was calculated using the G*Power, version 3.1.9.2 (Erdfelder, Faul, & Buchner, 1996, Kiel, Germany), considering the sample size of this study and α = 0.05, obtaining a statistical power (1 − β) of 0.96 for the performed analysis. The data were analyzed using SSPSS (Version 22.0; IBM Corp., Armonk, NY, USA), adopting a significance level of 5% (*p* = 0.05).

## Results

**Figure 1:**
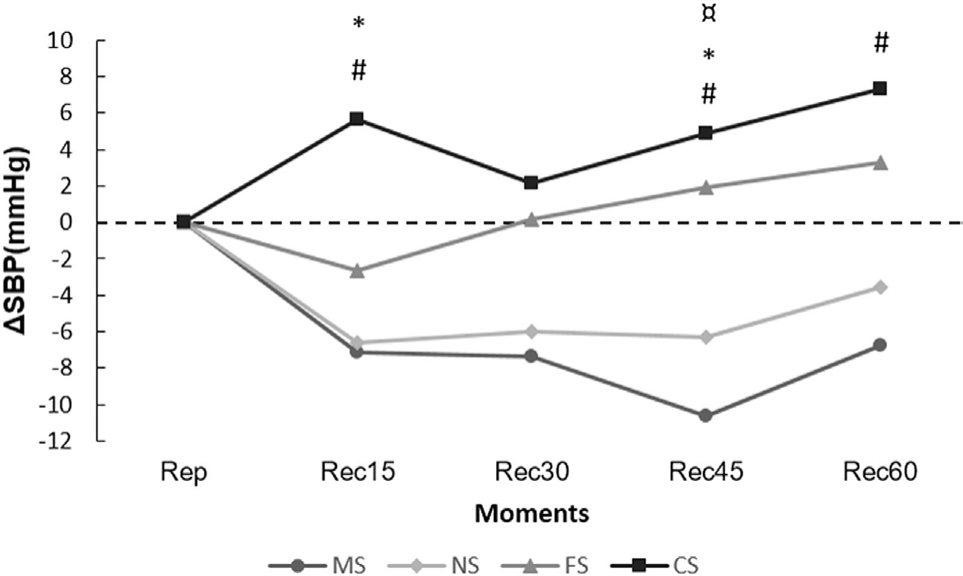
**Range of systolic blood pressure (ΔSBP) in the post-exercise moments**. ^#^*p* ≤ 0.05 [morning session (MS) vs. control session (CS)]; *p ≤ 0.05 [night session (NS) vs. CS)]; ^¤^*p* ≤ 0.05 [MS vs. fractionized session (FS)].

Figure 1 shows the SBP delta variations obtained in each post-exercise moments and at the control day. Reductions were observed (*p* ≤ 0.05) in Rec15 (−7.1 ± 12.1 vs. 5.6 ± 8.7 mmHg), Rec45 (−10.7 ± 12.9 vs. 4.9 ± 8.8 mmHg), and Rec60 (−6.8 ± 11.5 vs. ±7.3 ± 11.6 mmHg) moments when comparing morning session (MS) to control session (CS), and at the Rec45 (−10.7 ± 12.9 vs. 3.3 ± 9.5 mmHg) when MS was compared to FS. By analyzing the night session (NS), differences were found (*p* ≤ 0.05) only between Rec15 (−6.6 ± 7.7 vs. 5.6 ± 8.7 mmHg) and Rec45 (−6.3 ± 5.1 vs. 4.9 ± 8.8 mmHg) moments compared to CS.

**Figure 2:**
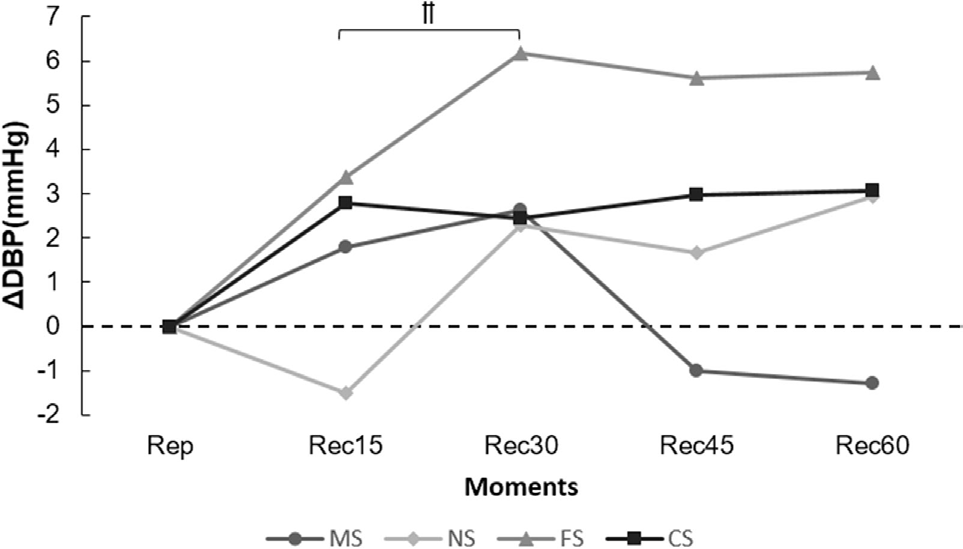
**Range of diastolic blood pressure (ΔDBP) in the post-exercise moments**. ^††^*p* ≤ 0.05 [Rec15 vs. Rec30 of night session (NS)].

Figure 2 presents the range of DBP expressed in the post-exercise moments. An atypical increase of BP occurred starting at Rec30 of NS (−1.5 ± 4.4 to 2.3 ± 4.2 mmHg); however, no differences were observed between sessions.

The percentage of variance from the acute response after CPT, taking CS as reference, is presented in Table 2. Only MS provided significant attenuation of the BP reactivity (*p* ≤ 0.05) compared to the CS, even NS and FS reducing the BP reactivity.

**Table 2:** **Variation of systolic blood pressure (SBP), diastolic blood pressure (DBP), and mean arterial pressure after the CPT**.

	CS (mmHg)	MS (mmHg) (%Δ)	NS (mmHg) (%Δ)	FS (mmHg) (%Δ)
CPT (SBP)	53.7	30.0 (−44.2)^#^	39.4 (−26.6)	39.5 (−26.5)
CPT (DBP)	28.4	11.7 (−58.8)^#^	19.3 (−32.1)	22.4 (−21.3)

*CPT, cold pressure test; CS, control session; MS, morning session; NS, night session; FS, fractionized session*.

*^#^*p* ≤ 0.05 (MS vs. CS)*.

*%Δ: in relation to CS values*.

## Discussion

The main findings of the present study were (a) the exercise session performed during the morning was more effective in promoting PEH for SBP than the other sessions; (b) the morning session was the only to promote attenuation of BP reactivity to SBP and DBP, compared to the CS; (c) the FS neither optimized PEH nor the attenuation of BP reactivity.

No differences were observed for the PEH for SBP between the MS and NS, yet de Brito et al. (32) observed higher PEH for SBP when 45 min of continuous AE session (50% of VO_2_peak) in pre-hypertensive was performed during the morning (−7 ± 3 mmHg) in relation to night (−3 ± 4 mmHg), on different days. Their finding may have occurred because these authors analyzed the effect of circadian rhythm on hemodynamic responses after continuous AE sessions in borderline hypertensive adults, unlike the present study, which aimed to investigate such effects in hypertensive middle-aged women after CE fractionized throughout the day.

The age and pathological condition of our volunteers as well as our exercise prescription may have influenced the results of the present study. The physiological changes caused by the aging process, the chronic presence of hypertension (55), and the influence caused by the pharmacological treatment (54) could reduce the statistical benefits of exercise for BP. Moreover, it is also known that PEH is a multifactorial phenomenon, influenced by biological (e.g., sex, age, ethnicity, and genetics), behavior (e.g., excessive alcohol consumption, smoking, low levels of physical activity, and hyper sodic and hyper caloric diets), and socioeconomic (e.g., purchasing power and educational level) factors (56–59).

The results for DBP in the present study did not show reductions after exercise sessions. It can be associated with the methodological aspects adopted since an indirect and subjective method for controlling the intensity of aerobic session was used, which may be underestimated the desired exercise intensity (moderate to severe), although the scale used is a validated instrument (45) and the volunteers have undergone the familiarization process.

The findings of the present study contrast with our hypothesis, since some studies showed that AE sessions performed throughout the day (3–4 sessions of 10 min) were associated with a better cardiovascular post-exercise response (14, 35–39), and probably linked to greater release of vasoactive substances from endothelial cells. However, we investigated the BP kinetic after a CE protocol performed during the morning and the night of the same day. Therefore, the execution of two stimuli associated with the presence of the resistance component may have inhibited the PEH phenomenon in the present study.

Furthermore, it is also known that other variables can influence the magnitude and duration of PEH, so understanding them is a key factor for the development of an effective physical exercise intervention. In this, some aspects can be highlighted as the ethnic and genetic factors (9), as well as the intensity (60), exercise volume (61), the type of recovery interval used between exercise series (53, 62, 63), the muscle mass recruited (64), and the physical exercise modality (18, 30).

Additionally, Keese et al. (18) demonstrated that the intensity of aerobic component in CE can influence the PEH, in which reported differences in the duration of the hypotensive effect to SBP after the sessions performed at 65 and 80% of VO_2_peak compared to the session performed at 50% of VO_2_peak in normotensive adults. Moreover, these authors demonstrated that the CE performed at 65 and 80% of VO_2_peak was able to promote greater PEH for DBP (~1.8 mmHg) when compared to the CS performed at 50% of VO_2_peak (−1.2 mmHg), and the highest intensity session provided greater PEH duration to DBP than other intensities. This factor can be related by higher plasma kallikrein activity and increased bioavailability of the nitric oxide at the post-exercise period, promoting greater vasodilation and consequently higher PEH for the more intense session (65).

For the BP reactivity, the MS was better to attenuate the BP reactivity compared to the day without exercise. Analyzing the change on BP after the CPT between MS and CS (MS_CPT_ − CS_CPT_), there were significant differences of −23.7 ± 16.0 and −16.7 ± 13.9 mmHg for SBP and DBP, respectively. In agreement with these findings, Moreira et al. (7) showed a cardioprotection state after a circuited exercise session by attenuating the BP reactivity at ~7 mmHg for SBP and at ~4 mmHg for DBP in adults of both sexes.

Despite the fact that significant BP attenuation was not observed after FS and NS for SBP and DBP in the present study (Table 2), the variation’s deltas in relation to CS were very high and, as previously discussed, it is extremely relevant as a cardioprotection effect. Considering the daily stress from nowadays and the physiological alterations related with hypertensive disease, all cardiovascular benefits obtained from these models of physical exercise are important.

All these data have great clinical and practical relevance, since sudden increases in BP favor the occurrence of some cardiovascular events, such as the rupture of atherosclerotic plaques, myocardial infarction, sudden cardiac death, and ischemic stroke (66). Complementary, Whelton et al. (17) showed that BP reductions of 5 mmHg are sufficient to reduce by 14% the cases of stroke mortality, 9% of cases of coronary heart disease, and 7% the total risk of mortality caused by coronary heart disease.

The present study has some limitations such as (a) the non-balance of volunteers by antihypertensive drug classes, such factor enables investigation, in fact, the interference of this variable in the PEH; (b) the non-utilization of a direct protocol for prescribing aerobic intensity; (c) the non-balance of the volunteers by the menopause stages, a feature that would allow analyzing the kinetic BP expressed from equivalent serum concentrations of estrogen hormones; (d) the non-standard regarding the drug administration schedule.

Despite the reported limitations, this study has high practical application, since most of the programs for control and treatment of hypertension, using physical exercise, concentrate their actions only in the aerobic component. Additionally, it is suggested that further studies based on the present proposal could be carried out, since due the daily duties, many people fail to practice physical activity for low availability of time, in this sense, the dissociation of a concurrent protocol seems to be an effective solution to maintain their health status preventing cardiovascular diseases. Moreover, applying new models of fractionated CEs to better understanding its cardiovascular benefits for hypertensive subjects must be investigated.

Additionally, the findings of the present study reinforce the security and benefits promoted by a physical exercise program performed following the American College of Sports Medicine (ACSM) guidelines, since significant acute reductions, as well as a cardioprotection state after the MS, were observed. It is important to highlight that the protocol adopted was performed around the inferior limit than suggested by ACSM (60–80% of 1-RM), and it was still effective to promote benefits in hypertensive middle-aged women.

In summary, the CE session proposed when performed in the morning is more effective for promoting PEH to SBP than the NS and FS, as well as showing greater attenuation of BP reactivity for SBP and DBP when compared to NS, FS, and CS in hypertensive middle-aged women. The NS also resulted some moments of PEH in relation to CS; however, there were no PEH for SBP after FS and for DBP after all sessions. Finally, the FS and NS provided good reductions, but not significant, for BP reactivity in relation to CS, with ~40% of reduction for SBP and ~20.5% of reduction for DBP.

## Author Contributions

LA, AS, and LS contributed to the design of the work, the acquisition, analysis, and interpretation of data, drafted the work; EP and JA contributed to the design of the work, the acquisition, analysis, and interpretation of data, drafted the work, and revisited it critically for important intellectual content; MF and RS contributed to the design of the work, the interpretation of data, revisited the work critically for important intellectual content. All authors approved the version to be published and agreed to be accountable for all aspects of the work in ensuring that questions related to the accuracy or integrity of any part of the work are appropriately investigated and resolved.

## Conflict of Interest Statement

The authors declare that the research was conducted in the absence of any commercial or financial relationships that could be construed as a potential conflict of interest.

## Abbreviations

8-RM, eight repetition maximum test; ACSM, American College of Sports Medicine; AE, aerobic exercise; BMI, body mass index; BP, blood pressure; CE, concurrent exercise; CPT, cold pressor test; CS, control session; DBP, diastolic blood pressure; DP, double product; FS, fractionized session; HC, hip circumference; HR, heart rate; ICC, intraclass correlation coefficient; MAP, mean arterial pressure; MS, morning session; NS, night session; PEH, post-exercise hypotension; RE, resistance exercise; SAH, systemic arterial hypertension; SBP, systolic blood pressure; SPE, subjective perception of effort; WC, waist circumference; WHO, World Health Organization; WHR, waist–hip ratio.
