# Supplementary material for: Fractionated Concurrent Exercise throughout the Day Does Not Promote Acute Blood Pressure Benefits in Hypertensive Middle-aged Women
Source: Front Cardiovasc Med. 2017 Feb 14;4:6. doi: 10.3389/fcvm.2017.00006 (PMC5308062; doi:10.3389/fcvm.2017.00006)
Supplement: Supplementary file 1 [file Table_1.DOC]

**Supplementary Table – Risk Factors and Antihypertensive drugs of the study sample (n=11).**

|  | **Category** | **n** | **%** |
| --- | --- | --- | --- |
| ***Risk Factors*** |  |  |  |
| Diabetes |  | 1 | 9,1 |
| Hyperthyroidism |  | 1 | 9,1 |
| Hypercholesterolemia |  | 7 | 63,6 |
| Body Mass Index (IMC) |  |  |  |
|  | *Normal* | 1 | 9,1 |
|  | *Overweight* | 2 | 18,2 |
|  | *Obese I* | 7 | 63,6 |
|  | *Obese II* | 1 | 9,1 |
|  |  |  |  |
| Infarction |  |  |  |
|  | *Pre-Infarction* | 2 | 18,2 |
|  | *Post-Infarction* | 1 | 9,1 |
|  |  |  |  |
| Menopause |  |  |  |
|  | *Present* | 3 | 27,3 |
|  | *Post Menopause* | 8 | 72,7 |
|  |  |  |  |
| Smoking |  |  |  |
|  | *Never Smoked* | 7 | 63,6 |
|  | *Ex-Smoker* | 4 | 36,4 |
|  |  |  |  |
| ***Antihypertensive Drugs*** |  |  |  |
| Diuretic |  | 5 | 45,5 |
| Angiotensin II receptor antagonists |  | 7 | 63,6 |
| Calcium channel blockers |  | 2 | 18,2 |
| β-blockers |  | 3 | 27,3 |
| Combined Drugs |  | 5 | 45,5 |
| Alternative Treatment ª |  | 1 | 9,1 |
|  |  |  |  |
| **Others Drugs** |  |  |  |
|  | *None* | 1 | 9,1 |
|  | *< 3 drugs* | 6 | 54,6 |
|  | *3 drugs* | 2 | 18,2 |
|  | *> 3 drugs* | 2 | 18,2 |

ª Teas or Eggplant Water
